# A Genetic Variant in miRNA-219-1 Is Associated with Risk of Esophageal Squamous Cell Carcinoma in Chinese Kazakhs

**DOI:** 10.1155/2015/541531

**Published:** 2015-08-24

**Authors:** Xiaoyue Song, Weiyan You, Jianbo Zhu, Xiaobin Cui, Jianming Hu, Yunzhao Chen, Wei Liu, Lianghai Wang, Shugang Li, Yutao Wei, Lan Yang, Feng Li

**Affiliations:** ^1^Department of Pathology and Key Laboratory for Xinjiang Endemic and Ethnic Diseases, Shihezi University School of Medicine, Shihezi, Xinjiang 832002, China; ^2^The First Affiliated Hospital, Shihezi University School of Medicine, Shihezi, Xinjiang 832002, China; ^3^Department of Preventive Medicine, Shihezi University School of Medicine, Shihezi, Xinjiang 832002, China

## Abstract

*Background*. Esophageal cancer (EC), an aggressive digestive tract malignancy, is one of the leading causes of cancer-related deaths worldwide. Besides environmental risk factors, genetic factors might play a key role in the EC carcinogenesis. The aim of the study is to evaluate the association of miR219-1 single-nucleotide polymorphisms (SNPs) with EC. *Methods*. A total of 248 Kazakh esophageal squamous cell carcinoma (ESCC) cases and 300 frequency-matched control subjects were recruited for this study. Genomic DNA was isolated from the samples. The miR-219-1 rs107822G > A and rs213210T > C genotypes were determined by matrix-assisted laser desorption/ionization time-of-flight mass spectrometry (MALDI-TOF MS). Linkage disequilibrium (LD) and haplotype analysis were used to detect the degree of association on miR-219-1 rs107822 and rs213210. Real-time quantitative polymerase chain reaction (qRT-PCR) was performed to detect miR-219-1 expression with miR-219-1 rs107822 polymorphism. *Result*. The SNP rs107822G > A in the miR-219-1 gene decreased the risk of Kazakh ESCC. Furthermore, two miR-219-1 SNPs, namely, rs107822 and rs213210, may tag each other to decrease the risk of Kazakh ESCC. These findings indicated that functional polymorphisms miR-219-1 rs107822G > A might change individual susceptibility to Kazakh ESCC.

## 1. Introduction

Esophageal cancer (EC), an aggressive digestive tract malignancy, is one of the leading causes of cancer-related deaths worldwide [1–5]. The two major histologic types of EC are esophageal squamous cell carcinoma (ESCC) and esophageal adenocarcinoma (EAC) [1, 3, 6–8]. ESCC is a predominant type of EC and ranks as the fourth primary cause of cancer-related deaths in China [2, 7, 9]. Most ESCC patients have already entered advanced stages of cancer before diagnosis, making the prognosis of ESCC patients ineffective in spite of the wide application of radical esophagectomy and systemic chemoradiotherapy, with overall survival rate of <10% and five-year postsurgical survival rate of 20% to 40% [2, 10–12].

The Kazakhs are one of the major minorities in China who are still living their traditional lifestyle with low resources and poor medical attention. The Kazakh population is characterized by increased ESCC-related incidence and mortality compared with other ethnic groups and general population of China [13, 14]. Therefore, identification of biological markers to improve early diagnosis rate, as well as development of gene therapies to reduce the ESCC mortality rate among Kazakhs, is very important for this ethnic group.

ESCC carcinogenesis is caused by multiple factors. The major significant risk factors for ESCC carcinogenesis include low consumption of fruits and vegetables, tobacco smoking, alcohol consumption, and even genetics [15–19].

Recent studies suggested that microRNAs (miRNAs) play important roles in ESCC carcinogenesis [20–22]. miRNAs are short noncoding RNA molecules that regulate gene expression by binding with the 3′ untranslated region (3′ UTR) of their target genes, leading to mRNA degradation or translational repression. miRNAs regulate a wide range of biological processes, including cell-cycle control, differentiation, cell proliferation, and apoptosis [20–22]. miRNAs also function as tumor suppressor or oncogenes participating in diverse biological pathways. Thus, the loss or gain of function of a specific miRNA represents a vital event in tumorigenesis [20–22].

Single-nucleotide polymorphisms (SNPs) in pre-miRNA or miRNA sequence affect the susceptibility of numerous cancers. For example, polymorphism rs11614913T > C in hsa-miR-196a2 was associated with the increased risk of gastric cancer [23]. Polymorphism rs6505162C > A in miR-423 decreased the risk of Caucasian EC [24], whereas polymorphism rs2910164G > C in miR-146a precursor contributed to breast cancer and hepatocellular carcinoma susceptibility [25, 26]. The rs213210 genetic variant in miR-219-1 significantly increased the mortality risk of stage III colorectal adenocarcinoma patients and was associated with the increased risk of Caucasian EC; however, no significant correlation was found between rs213210 in miR-219-1 and bladder cancer and renal cell carcinoma [24, 27–29]. Furthermore, the exact function of miR-219-1 during tumorigenesis remains unclear. SNP frequencies vary among different ethnic groups, regions, and backgrounds. Thus, studying the polymorphism in the miR-219-1 gene sequence for Kazakh ESCC will provide new insights into the pathogenesis of this cancer.

## 2. Materials and Methods

### 2.1. Study Population

The study population consisted of 248 ESCC patients and 300 cancer-free controls. All samples were recruited from the First Affiliated Hospital of Shihezi University, People's Hospital of XinJiang Uygur Autonomous Region and the Xinjiang Yili Prefecture Friendship Hospital between 1984 and 2012. Up to 248 patients diagnosed with histopathologically confirmed ESCC were randomly recruited for this study. No constraints regarding age, sex, and cancer stage were implemented, but patients who previously had other cancers (including any metastasized cancer), undergone surgery (other than diagnostic biopsies), chemotherapy, or radiotherapy before recruitment, or had blood transfusion in the preceding six months were excluded from the study. We collected data on clinical and pathological variables, such as tumor site, invasion depth, and distant metastasis, from the medical records of the patients. The differentiation grade, TNM stage, and lymph node status were classified in accordance with the American Joint Committee on Cancer (AJCC/UICC) TNM classification (seventh edition) (Table 1).

The 300 control subjects were healthy individuals without any cancer and hereditary disease which were matched to the cases in terms of age (±5 years), gender, and ethnicity of patients who visited outpatient clinics as part of an early detection and treatment of EC project. Each subject was individually questioned by trained interviewers using a pretested questionnaire to collect information on demographics (including age and sex), related risk factors (including tobacco smoking, alcohol consumption, fruit and vegetable consumption, and irregular eating diet), and medical history (digestive disease history and family history of EC). After the interview, 5 mL of venous blood was collected from each subject by using Vacutainer tubes containing EDTA; samples were stored at −80°C. All specimens were collected with consent of inspectors. The Institutional Ethics Review Board of the First Affiliated Hospital of Shihezi University School of Medicine approved this study.

### 2.2. DNA Isolation and Genotyping

Genomic DNA from whole blood or ESCC tissues was extracted using QIAamp DNA Mini Kit (QIAGEN, Valencia, CA, USA) in accordance with the manufacturer's protocol. The quantity and quality of DNA were determined using NanoDrop Spectrophotometer (ND-1000). As an internal control, all purified genomic DNA samples were amplified by polymerase chain reaction (PCR) following the manufacturer's recommendations. Site-specific PCR and detection primers were designed using MassARRAY Assay Design 3.0 software (Sequenom Inc., San Diego, CA, USA). SNP genotyping was performed using the Sequenom MassARRAY platform (Sequenom Inc., San Diego, CA, USA) with matrix-assisted laser desorption/ionization time-of-flight mass spectrometry (MALDI-TOF MS) at the Key Laboratories for Xing Jiang Endemic and Ethnic Diseases (Shihezi University School of Medicine). Genotyping was performed by two people in a blinded fashion, with 10% of the samples randomly selected for repeated analyses. The results were 100% consistent.

### 2.3. RNA Isolation and Real-Time Quantitative Polymerase Chain Reaction (qRT-PCR)

Total RNA was extracted from EC tissues or adjacent normal tissues using RNeasy FFPE Kit (QIAGEN, Hilden, Germany) following the manufacturer's protocol (*n* = 100). qRT-PCR was performed using SYBR Green PCR Master Mix (QIAGEN, Hilden, Germany) containing ROX as a reference dye. Normalization was performed with small nuclear RNA U6 as universally expressed endogenous control (Applied Sangon Biotech Co., Ltd., Shanghai, China). miR-219-1-5p expression levels were calculated using the 2^(−ΔCT)^ values provided by the manufacturer. All qRT-PCR reactions were performed in triplicate. Expression data was analyzed with GraphPad Prism (version 5.01).

### 2.4. Statistical Analysis

All statistical analyses were performed using Statistical Products and Services Solutions (SPSS) software version 17.0 (U.S.). Hardy-Weinberg equilibrium (HWE) was performed by a goodness-of-fit *χ* ^2^ test to compare the observed genotype frequencies with those of the expected genotype among the controls. Differences in the distributions of demographic characteristics, genotype of the miR219-1 rs107822G > A, and rs213210T > C variants between the cases and controls were analyzed using Student's *t*-test and *χ* ^2^ test. The associations between the two SNPs in miR-219-1 and the risk of ESCC were evaluated by computing the odds ratios (ORs) at 95% confidence intervals (CIs) using logistic regression analyses for crude ORs and adjusted ORs when adjusting for age and sex. Haplotype frequencies and linkage disequilibrium (LD) measurements were obtained using SHEsis Software (online version: http://analysis.bio-x.cn/SHEsisMain.htm) based on the observed genotypes. Statistical comparisons of the relative expression of mature miR-219-1 among the different genotypes in ESCC tissues were performed using paired-sample *t*-test or two-sample *t*-test. All statistical analyses were two-sided, and *P* < 0.05 was considered statistically significant.

## 3. Results

### 3.1. Characteristics of the Study Population

Table 1 summarizes the characteristics of the 248 cases and 300 controls included in this study. The age was 56.23 ± 9.02 (mean ± SD) years for ESCC cases and 55.16 ± 10.62 (mean ± SD) years for control. The cases and controls adequately matched the age and sex suggested by Student's *t*-test and *χ* ^2^ test results. No significant difference was observed for age and gender (*P* = 0.212 and *P* = 0.212, resp.). The case group included 47 (19.0%) well-differentiated patients, 47 (19.0%) moderately differentiated patients, 49 (19.8%) poorly differentiated patients, 98 (42.2%) T1/T2 stage patients, 134 (57.8%) T3/T4 stage patients, 132 (53.2%) patients without lymph node metastasis, 116 (46.8%) patients with lymph node metastasis, 154 (64.7%) TNM stages I-II patients, and 84 (35.3%) TNM stages III-IV patients. The primary information for the two SNPs of miR-219-1 is presented in Table 2. The observed genotype frequencies for miR-219-1 rs107822G > A and rs213210T > C were all consistent with the HWE in controls (*P* = 0.323 and *P* = 0.954, resp.).

### 3.2. Associations between miR-219-1 rs107822G > A and rs213210T > C Polymorphisms and the Risk of Kazakh ESCC

The genotype distributions of miR219-1 rs107822G > A and rs213210T > C in the cases and controls are listed in Table 3. In the single-locus analyses, the genotype frequencies of miR-219-1 rs107822G > A were 32.8% (GG), 49.5% (GA), and 17.7% (AA) in the case patients and 24.6% (GG), 38.2% (GA), and 37.2% (AA) in the controls. A significant difference was observed between the cases and controls (*P* = 2.606 × 10^−5^, Table 3). The A allele reduced the risk of Kazakh ESCC compared with the G allele (OR = 0.573, 95% CI = 0.441–0.744, *P* = 2.837 × 10^−5^, Table 3). When the miR-219-1 rs107822 GG homozygote genotype was used as reference group in the codominant model, the GA genotypes were not associated with the risk for Kazakh ESCC (GA versus GG: adjust OR = 0.976, 95% CI = 0.626–1.522, *P* = 0.914, Table 3), but the AA genotype showed a statistically decreased risk for Kazakh ESCC (AA versus GG: adjust OR = 0.365, 95% CI = 0.217–0.614, *P* = 1.429 × 10^−4^, Table 3). In the recessive model, the miR-219-1 rs107822 AA homozygote genotype was associated with a statistically decreased risk for Kazakh ESCC, compared with the miR-219-1 rs107822 GG + GA genotypes (AA versus GG + GA: adjust OR = 0.371, 95% CI = 0.238–0.577, *P* = 1.134 × 10^−5^, Table 3). However, in the dominant model, the miR-219-1 rs107822 GA + AA variants were not associated with the risk for Kazakh ESCC, compared with the miR-219-1 rs107822 GG genotype (GA + AA versus GG: adjust OR = 0.677, 95% CI = 0.449 to 1.020, *P* = 0.063, Table 3).

No association was observed between the miR-219-1 rs213210T > C polymorphisms and the risk of Kazakh ESCC (*P* > 0.05) (Table 3).

### 3.3. Correlations of Clinicopathological Parameters and miR-219-1 Polymorphism in Kazakh Patients with ESCC

Stratification analyses were performed to further investigate the potential effects of miR-219-1 rs107822G > A genotype on Kazakh ESCC risk in terms of clinicopathological parameters (including gender, age, histologic stage, depth of invasion, lymph node metastasis, and TNM stage). However, no significant association was found between miR-219-1 rs107822G > A and Kazakh ESCC concerning gender, age, histologic stage, depth of invasion, lymph node metastasis, and TNM stage (*P* > 0.05 for all) (Table 4).

### 3.4. Linkage Disequilibrium and Haplotype Analysis of miR219-1 SNPs

**Figure 1:**
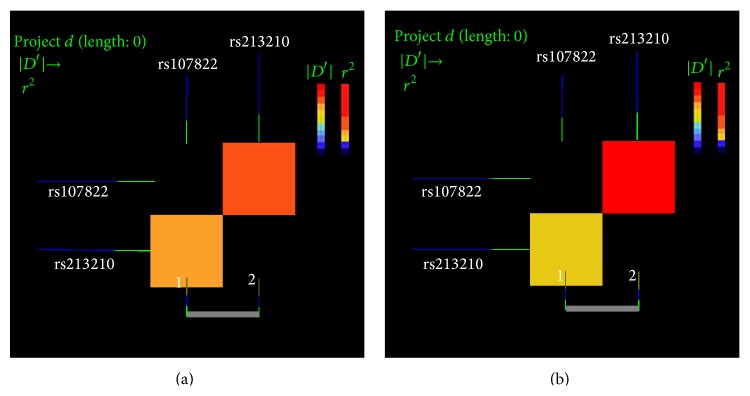
Linkage disquilibrium graph of the two SNPs, rs107822 and rs213210, in the miR-219-1 gene in the Kazakh population. The linkage disequilibrium values were caculated using *r* ^2^ and *D*′ statistic (http://analysis.bio-x.cn/SHEsisMain.htm). (a) Linkage disequilibrium analysis in the cases group (coefficient *D*′ = 0.8808, coefficient *r* ^2^ = 0.2503). (b) Linkage disequilibrium analysis in the controls group (coefficient *D*′ = 1, coefficient *r* ^2^ = 0.1732). Areas with red blocks represent stronger linkage disequilibrium.

The LD of rs107822 and rs213210 in miR-219-1 was estimated using *r* ^2^ and *D*′ statistic (http://analysis.bio-x.cn/SHEsisMain.htm). Two miR219-1 polymorphisms, namely, rs107822 and rs213210, showed strong LD in the cases and complete LD in the controls (cases: *D*′ = 0.8808, *r* ^2^ = 0.2503; controls: *D*′ = 1, *r* ^2^ = 0.1732; Figure 1), suggesting that the two miR-219-1 SNPs may tag each other for Kazakh ESCC. Haplotype analysis was performed to further evaluate the combined effects of the two miR-219-1 polymorphisms on the risk of Kazakh ESCC. Among the haplotypes derived from the observed genotypes, the G_rs107822_T_rs213210_ was the most common in both cases and controls (56.6% and 45.4%, resp.) (Table 5). Individuals with the haplotypes G_rs107822_T_rs213210_ and A_rs107822_T_rs213210_ were associated with an increased risk of Kazakh ESCC (*P* = 4.970 × 10^−4^, OR = 1.597, 95% CI = 1.227 to 2.080; *P* = 7.720 × 10^−5^, OR = 0.559, 95% CI = 0.419–0.747, resp.). None of the other haplotypes was significantly associated with the risk of Kazakh ESCC (*P* > 0.05).

### 3.5. Effect of miR-219-1 Polymorphisms on Its Expression

**Figure 2:**
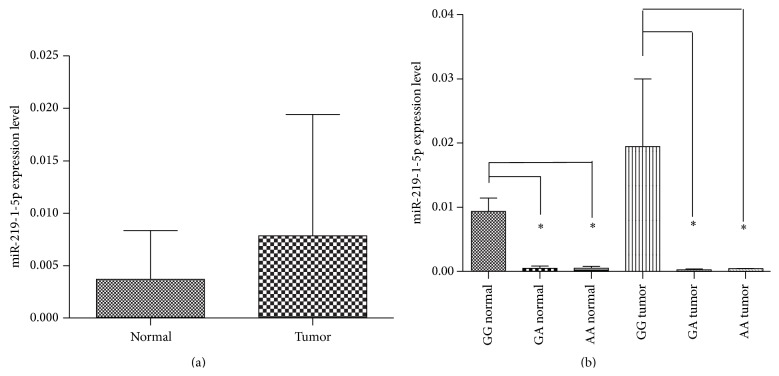
miR-219-1-5p expression level in ESCC tissues. (a) miR-219-1-5p levels were determined in normal tissue and tumor tissue samples from ESCC patients (*N* = 100) by qRT-PCR. All values were normalized to U6 RNA levels. Mean expression levels were 3.732 × 10^−3^ in normal tissue sample and 7.840 × 10^−3^ in tumor tissue sample (*P* = 0.287). (b) miR-219-1-5p levels were determined for different genotype in normal tissue and tumor tissue samples from ESCC patients (*N* = 100). All values were normalized to U6 RNA levels. Mean expression levels for rs107822 genotypes are 9.4 × 10^−3^ (GG, normal), 4.0 × 10^−4^ (GA, normal), and 5.0 × 10^−4^ (AA, normal) and 2.1 × 10^−2^ (GG, tumor), 3.0 × 10^−4^ (GA, tumor), and 4.0 × 10^−4^ (AA, tumor). *P*_GG-GA normal_ = 1.3 × 10^−4^, *P*_GG-AA normal_ = 0.001; *P*_GG-GA tumor_ = 0.004, *P*_GG-AA tumor_ = 0.012.

To study the effect of the variants of miR-219-1 rs107822 on mature miRNA expression, qRT-PCR was used to measure the miR-219-1-5p expression levels with different rs107822 genotypes in tumor and normal tissue samples (*n* = 100). We found that miR-219-1-5p expression with GG genotype is higher than GA and AA genotypes in both tumor and normal tissues (mean_GG normal_ = 9.4 × 10^−3^, mean_GA normal_ = 4.0 × 10^−4^, mean_AA normal_ = 5.0 × 10^−4^; mean_GG tumor_ = 2.1 × 10^−2^, mean_GA tumor_ = 3.0 × 10^−4^, mean_AA tumor_ = 4.0 × 10^−4^, *P*_GG-GA normal_ = 1.3 × 10^−4^, *P*_GG-AA normal_ = 0.001, *P*_GG-GA tumor_ = 0.004, and *P*_GG-GA tumor_ = 0.012). However, the mature miR-219-1 expression showed no difference between the tumor tissues and the adjacent normal tissues (mean_normal_ = 3.732 × 10^−3^, mean_tumor_ = 7.840 × 10^−3^, *P* = 0.287) (Figure 2).

## 4. Discussion

Carcinogenesis and development of ESCC are complex biological processes involving multiple factors, including environmental risk factors. ESCC is a polygenic hereditary disease that can also be influenced by genetic factors [5, 14, 30, 31]. Along with other researchers, we have long carried on the etiological and epidemiological investigation of numerous aspects of the high Kazakh ESCC incidence rate, which may include traditional habits and customs, such as eating hot meals, hard foods, yogurt knots, and tea; smoking; lack of folic acid; natural geographical environment [31]; genetic factors, such as PLCE1, hRFT1, and ECRG2 [14, 32, 33]; and microbial factors, such as HPV [34]. These factors are associated with the etiology and pathogenesis of Kazakh ESCC, but the main mechanisms regulating the development of Kazakh ESCC remain unclear.

SNP in miRNA genes, a common type of genetic variation in the human genome, was associated with different types of cancer susceptibility [5, 15, 26, 35], as SNP can play a predictive role in the risk of cancer development and clarify the pathophysiological mechanism of carcinogenesis. Several studies have linked the miR-219-1 SNP to cancer susceptibility [24, 27–29].

miR-219-1, which is located on chromosome 6p21.32 and initially described as a brain-specific miRNA, is a clock- and light-regulated gene that controls the length of the circadian cycle [36], as this miRNA is a vital regulator of oligodendrocyte differentiation [37, 38]. A subsequent research involving zebrafish discovered that overexpression of miR-219-1 can induce embryonic cell death [39]. Recent research found out that miR-219 might play an important role in tumorigenesis as this miRNA was shown to decrease in different types of cancer [40, 41]. Some case-control studies concentrated on the relationship between the miR-219-1 polymorphism and cancer risk [24, 27–29]. Interestingly, miR-219-1 rs213210 has significantly increased the mortality risk of confirmed stage III colorectal adenocarcinoma patients [27] and increased the risk of Caucasian EC [24], indicating that miR-219-1 polymorphisms may contribute to cancer susceptibility. However, the relationship between miR-219-1 polymorphism and Kazakh ESCC risk has never been reported.

In the present case-control study, we evaluated the association between miR-219-1 polymorphism and the risk of Kazakh ESCC. In the codominant and recessive models, miR-219-1 rs107822G > A polymorphism was associated with significantly decreased Kazakh ESCC risk. However, no significant association existed between miR-219-1 rs213210T > C and the risk of Kazakh ESCC in our study. These results suggested that the polymorphism rs107822G > A in the miR-219-1 is a protective factor against ESCC in the Kazakh population. Our stratified analyses found no significant association between miR-219-1 rs107822 and Kazakh ESCC with regard to gender, age, histologic stage, depth of invasion, lymph node metastasis, and TNM stage. However, our findings are not consistent with previous results [24, 27], possibly because the same variation in miRNA plays varying roles in diverse types of cancers, and the difference in ethnicity, regions, and background of population might have led to different consequences.

Furthermore, two SNPs, rs107822 and rs213210, in the miR-219-1 gene showed strong LD with the cases and complete LD with the controls. Haplotype analysis results showed that the G_rs107822_T_rs213210_ haplotype is a risk factor and A_rs107822_T_rs213210_ haplotype is a protective factor associated with EC in Xinjiang Kazakh, indicating that the two SNPs in the miR-219-1 may tag each other to decrease the risk of Kazakh ESCC. Furthermore, polymorphisms with miR-219-1 can be used as candidate biomarkers for cancer susceptibility.

miRNAs, a class of novel posttranscriptional regulators, regulate almost one-third of human genes. miRNA-related SNPs can influence the miRNA functions through different mechanisms [5, 21, 42]. First, miRNAs can change gene transcription. Artificial mutations in the terminal loop of miRNAs, such as miR-31 and miR-30, can block miRNA maturation [43]. Sun et al. found that an SNP within the miR-27a terminal loop significantly influenced the miR-27a genotype-based expression [44], suggesting that the SNPs located in the miRNA terminal loop may slightly influence the processing of pri-miRNA and affect the miRNA biogenesis. Second, through interfering with pri-miRNA and pre-miRNA processing and maturation, an SNP located at nucleotide 8 (t8) of mature miR-125a in a normal subject blocks pri-miR-125a processing to pre-miRNA, thereby reducing the miRNA-mediated translational suppression of its target [45]. rs11614913 CC in hsa-mir-196a2 was associated with a statistically significant increase in mature hsa-mir-196a expression [46], suggesting that miRNA-related SNPs may affect pri-miRNA to pre-miRNA processing and maturation. Lastly, alteration of the miRNA-mRNA interaction affinity, such as rs11614913 SNP in hsa-mir-196a2, can affect the binding of mature hsa-mir-196a2-3p to its target LSP1Mrna [45, 46], suggesting that SNPs can affect the miRNA functions by changing the miRNA-mRNA interaction. However, several studies showed that SNPs in pre-miRNA are related to cancer risk but do not alter the expression levels of the mature miRNA. The rs6505162A > C in pre-miRNA miR-423 increased the EC risk but did not change the levels of mature miR-423 [5].

To further characterize the functional relevance of the miR219-1 rs107822 SNP, qRT-PCR was used to detect the effects of different SNP rs107822 genotypes on the expression of mature miR-219-1. The miR-219-1 rs107822 GA and AA genotypes decreased the mature miR-219-1 expression compared with the miR-219-1 rs107822 GG genotypes in both Kazakh ESCC tumor tissues and adjacent normal tissues. However, the mature miR-219-1 expression showed no difference between the Kazakh ESCC tumor tissues and adjacent normal tissues, which is inconsistent with a previous report wherein miR-219-1 expression was decreased in different types of cancer [40, 41]. miRNAs regulate gene expression by binding with the 3′ UTR of their target genes. Our results showed no difference between the expression of miR-219-1 in the tumor tissues and the adjacent normal tissues. However, the miR-219-1 rs107822G > A polymorphisms decreased the risk of Kazakh ESCC. Thus, we deduced that miR-219-1 rs107822G > A polymorphisms may be ascribed to the binding efficiency between miR-219-1 and its target mRNAs before influencing miR-219-1 to regulate its target mRNAs and decrease the risk of Kazakh ESCC. However, further studies are required to confirm this hypothesis.

Several limitations of this case-control study need to be addressed. First, given that the cases in our study were enrolled from hospitals, and controls were enrolled from an early detection and treatment of EC project, the samples may not represent the general population. However, we selected the cases and controls following the strict selection criteria and genotype distribution of the controls in HWE. Second, the sample size was relatively small, particularly for stratified analyses evaluating the relationship between the amount of miR-219-1 and clinicopathological parameters of Kazakh ESCC patients. These results should be explained thoroughly with further studies using a larger sample size to confirm the current findings and clarify the effects of the polymorphism expression of miR-219-1 on the carcinogenesis of Kazakh ESCC. Nevertheless, our findings provided valuable insights and interesting information that can guide future studies in this area.

## 5. Conclusions

In conclusion, the present study provided evidence that the SNP rs107822G > A in miRNA-219-1 gene decreased the risk of Kazakh ESCC. Furthermore, two SNPs (rs107822 and rs213210) in the miR-219-1 gene may tag each other to decrease the risk of Kazakh ESCC. miR-219-1 rs107822G > A influenced the mature miR-219-1 expression with different rs107822 genotypes in both Kazakh ESCC tumor tissues and adjacent normal tissues. However, mature miR-219-1 levels showed no difference between the Kazakh ESCC tumor tissues and the adjacent normal tissues.

## Figures and Tables

**Table 1:** Characteristics of ESCC cases and controls.

Variables	Cases [*n* (%)]	Controls [*n* (%)]	*P* ^a^ value
	*n* = 248	*n* = 300	
Age (years), mean ± SD	56.23 (±9.02)	55.16 (±10.62)	0.212^b^
Age (years)			0.334
<57	122 (49.2)	160 (53.3)	
≥57	126 (50.8)	140 (46.7)	
Sex			0.212
Male	143 (57.7)	157 (52.3)	
Female	105 (42.3)	143 (47.7)	
Differentiation status			
Well-differentiated	47 (19.0)		
Moderately differentiated	152 (61.2)		
Poorly differentiated	49 (19.8)		
Tumor location			
Upper	14 (5.6)		
Middle + lower	234 (94.4)		
Depth of invasion			
T1/T2	98 (42.2)		
T3/T4	134 (57.8)		
Lymph node metastasis			
No	132 (53.2)		
Yes	116 (46.8)		
Tumor node metastasis stage			
I-II	154 (64.7)		
III-IV	84 (35.3)		

^a^Two-sided  *χ* ^2^ test.

^b^Student's *t*-test.

**Table 2:** Primary information for miR-219-1 polymorphisms.

Gene	Genotyped SNPs	Chromosome	MAF^a^ for Chinese in database	MAF in our controls	*P* value for HWE^b^ test in our controls	RefSNP alleles	Ancestral allele
miR219-1	rs107822	6	0.433	0.492	0.323	A/G	G
	rs213210	6	0.422	0.170	0.954	C/T	T

^a^MAF: minor allele frequency; ^b^HWE: Hardy-Weinberg equilibrium.

**Table 3:** Logistic regression analyses of associations between miR-219-1 rs107822G > A and rs213210T > C polymorphisms and risk of Kazakh ESCC.

Genotype	Cases [*n* (%)]	Controls [*n* (%)]	Crude OR (95% CI)	*P* value	Adjusted OR^a^ (95% CI)	*P* value
	(*n* = 248)	(*n* = 300)				
miR-219-1 (rs107822G > A)						
GG	65 (32.8)	67 (24.6)	1		1	
GA	98 (49.5)	104 (38.2)	0.971 (0.626–1.506)	0.897	0.976 (0.626–1.522)	0.914
AA	35 (17.7)	101 (37.2)	**0.357 (0.214**–**0.597)**	**8.662 × 10** ^−**5**^	**0.365 (0.217**–**0.614)**	**1.429 × 10** ^−**4**^
GG vs. GA vs. AA						**2.606 × 10** ^−**5**^
GA+AA	133 (67.2)	205 (75.4)	0.6669 (0.446–1.003)	0.052	0.677 (0.449–1.021)	0.063
GG+GA	163 (82.3)	171 (62.8)	1		1	
AA	35 (17.7)	101 (37.2)	**0.364 (0.234**–**0.565)**	**6.646 × 10** ^−**6**^	**0.371 (0.238**–**0.577)**	** 1.134 × 10** ^−**5**^
G allele	228 (57.6)	238 (43.8)	1			
A allele	168 (42.4)	306 (56.2)	**0.573 (0.441**–**0.744)**	**2.837 × 10** ^−**5**^		
miR-219-1 (rs213210T > C)						
TT	161 (65.7)	195 (68.4)	1		1	
CT	78 (31.8)	83 (29.2)	1.138 (0.784–1.653)	0.496	1.172 (0.805–1.709)	0.408
CC	6 (2.5)	7 (2.4)	1.038 (0.342–3.151)	0.947	1.057 (0.346–3.231)	0.923
TT vs. CT vs. CC						0.793
CT+CC	84 (34.3)	90 (31.6)	1.130 (0.786–1.626)	0.508	1.164 (0.806–1.680)	0.419
TT+TC	259 (97.5)	278 (97.6)	1		1	
CC	6 (2.5)	7 (2.4)	0.997 (0.331–3.007)	0.996	1.003 (0.330–3.044)	0.996
T allele	400 (81.6)	473 (83)	1			
C allele	90 (18.4)	97 (17)	1.097 (0.800–1.505)	0.565		

^a^Logistic regression adjusted for age and sex.

*P* values under 0.05 were indicated in bold font.

**Table 4:** Stratification analyses between miR-219-1 rs107822G > A polymorphism and clinicopathological parameters of Kazakh ESCC patients.

Parameter	GG	GA	AA	GG	GA		AA		GA+AA		GG+GA	AA	
	Case/control				Adjusted OR (95% CI)	*P *	Adjusted OR (95% CI)	*P *	Adjusted OR (95% CI)	*P*		Adjusted OR (95% CI)	*P* value
Gender^a^													
Male	36/47	51/51	25/41	1.00	1.306 (0.729–2.337)	0.369	0.796 (0.411–1.540)	0.498	1.079 (0.635–1.832)	0.780	1.00	0.687 (0.386–1.221)	0.199
Female	19/30	37/53	30/50	1.00	1.102 (0.541–2.246)	0.789	0.947 (0.456–1.969)	0.885	1.027 (0.535–1.971)	0.936	1.00	0.889 (0.505–1.565)	0.684
Age^a^													
<57	27/45	33/54	28/48	1.00	0.968 (0.514–1.822)	0.920	0.715 (0.313–1.631)	0.424	0.893 (0.491–1.624)	0.711	1.00	0.737 (0.354–1.532)	0.413
≥57	38/32	45/50	27/43	1.00	0.758 (0.408–1.408)	0.380	0.529 (0.270–1.036)	0.062	0.652 (0.372–1.144)	0.135	1.00	0.620 (0.351–1.087)	0.099
Histologic grade^b^													
Well-differentiated	12	15	5	1.00	1.345 (0.569–3.184)	0.500	1.573 (0.49–5.051)	0.447	1.401 (0.619–3.170)	0.419	1.00	1.312 (0.460–3.742)	0.611
Moderately/poorly differentiated	53	83	30										
Depth of invasion^b^													
T1+T2	16	33	13	1.00	0.603 (0.289–1.258)	0.178	0.461 (0.180–1.183)	0.107	0.564 (0.279–1.141)	0.111	1.00	0.641 (0.288–1.427)	0.276
T3+T4	44	59	19										
Lymph node metastasis^b^													
No	34	47	23	1.00	1.261 (0.665–2.393)	0.477	0.620 (0.261–1.475)	0.280	1.058 (0.576–1.945)	0.856	1.00	0.537 (0.249–1.158)	0.113
Yes	31	51	12										
TNM stage^b^													
I+II	38	58	19	1.00	1.075 (0.548–2.111)	0.833	1.234 (0.505–3.015)	0.644	1.113 (0.586–2.114)	0.744	1.00	1.180 (0.537–2.592)	0.681
III+IV	24	36	13										

^a^Stratification analysis to evaluate the effects of variant genotypes on the risk of ESCC by age and sex.

^b^Logistic regression analysis for the effects of miR-219-1 variants on risk of Kazakh ESCC with different histologic grade, depth of invasion, lymph node metastasis, and clinical stage.

**Table 5:** Distribution of miR-219-1 haplotypes and their association with Kazakh ESCC.

Haplotypes	Haplotype frequencies^a^		OR (95% CI )	*χ* ^2^	*P* ^b^
	Cases [(*n*%)]	Controls [(*n*%)]			
	(*n* = 248)	(*n* = 300)			
AC	70 (17.8)	90 (17.2)	1.049 (0.744–1.480)	0.075	0.78
AT	98 (24.8)	195 (37.4)	0.559 (0.419–0.747)	15.645	7.72 × 10^−5^
GC	3 (0.7)	0 (0.00)	—	—	—
GT	223 (56.6)	237 (45.4)	1.597 (1.227–2.080)	12.139	4.97 × 10^−4^

The combination order of SNP is as follows: rs107822G/A, rs213210 C/T.

^a^Only haplotypes with frequencies of ≥0.03% are shown.

^b^ *P* value for difference in haplotype frequencies between cases and control.

Haplotype frequencies were obtained using SHEsis Software (http://analysis.bio-x.cn/SHEsisMain.htm).

## Abbreviations

EC: Esophageal cancer
ESCC: Esophageal squamous cell carcinoma
EAC: Esophageal adenocarcinoma
MALDI-TOF MS: Matrix-assisted laser desorption/ionization time-of-flight mass spectrometry
LD: Linkage disequilibrium
qRT-PCR: Real-time quantitative polymerase chain reaction
PCR: Polymerase chain reaction
3′ UTR: 3′ Untranslated region
SNPs: Single-nucleotide polymorphisms
IERB: Institutional Ethics Review Board
AJCC/UICC: American Joint Committee on Cancer
ND-1000: NanoDrop Spectrophotometer
HWE: Hardy-Weinberg equilibrium.

## References

[1] Pisani P., Parkin D. M., Bray F., Ferlay J. (1999). Estimates of the worldwide mortality from 25 cancers in 1990. *International Journal of Cancer*.

[2] Li H., Zheng D., Zhang B. (2014). Mir-208 promotes cell proliferation by repressing SOX6 expression in human esophageal squamous cell carcinoma. *Journal of Translational Medicine*.

[3] Enzinger P. C., Mayer R. J. (2003). Esophageal cancer. *The New England Journal of Medicine*.

[4] Wheeler J. B., Reed C. E. (2012). Epidemiology of esophageal cancer. *Surgical Clinics of North America*.

[5] Wang Y., Vogelsang M., Schäfer G., Matejcic M., Parker M. I. (2013). MicroRNA polymorphisms and environmental smoke exposure as risk factors for oesophageal squamous cell carcinoma. *PLoS ONE*.

[6] Umar S. B., Fleischer D. E. (2008). Esophageal cancer: epidemiology, pathogenesis and prevention. *Nature Clinical Practice Gastroenterology & Hepatology*.

[7] Zhang B.-J., Gong H.-Y., Zheng F., Liu D.-J., Liu H.-X. (2014). Up-regulation of miR-335 predicts a favorable prognosis in esophageal squamous cell carcinoma. *International Journal of Clinical and Experimental Pathology*.

[8] Vizcaino A. P., Moreno V., Lambert R., Parkin D. M. (2002). Time trends incidence of both major histologic types of esophageal carcinomas in selected countries, 1973–1995. *International Journal of Cancer*.

[9] Xu Y., Yu X., Chen Q., Mao W. (2012). Neoadjuvant versus adjuvant treatment: which one is better for resectable esophageal squamous cell carcinoma?. *World Journal of Surgical Oncology*.

[10] Siegel R., Ma J., Zou Z., Jemal A. (2014). Cancer statistics, 2014. *CA—Cancer Journal for Clinicians*.

[11] Xing D., Tan W., Lin D. (2003). Genetic polymorphisms and susceptibility to esophageal cancer among Chinese population (Review). *Oncology Reports*.

[12] Lee K.-H., Goan Y.-G., Hsiao M. (2009). MicroRNA-373 (miR-373) post-transcriptionally regulates large tumor suppressor, homolog 2 (LATS2) and stimulates proliferation in human esophageal cancer. *Experimental Cell Research*.

[13] Lu J.-B., Yang W.-X., Liu J.-M., Li Y. S., Qin Y. M. (1985). Trends in morbidity and mortality for oesophageal cancer in Linxian county, 1959–1983. *International Journal of Cancer*.

[14] Cui X.-B., Chen Y.-Z., Pang X.-L. (2013). Multiple polymorphisms within the PLCE1 are associated with esophageal cancer via promoting the gene expression in a Chinese Kazakh population. *Gene*.

[15] Zhang J., Huang X., Xiao J. (2014). Pri-miR-124 rs531564 and pri-miR-34b/c rs4938723 polymorphisms are associated with decreased risk of esophageal squamous cell carcinoma in Chinese populations. *PLoS ONE*.

[16] Yu M. C., Garabrant D. H., Peters J. M., Mack T. M. (1988). Tobacco, alcohol, diet, occupation, and carcinoma of the esophagus. *Cancer Research*.

[17] Smith K. J., O'Brien S. M., Smithers B. M. (2005). Interactions among smoking, obesity, and symptoms of acid reflux in Barrett's esophagus. *Cancer Epidemiology Biomarkers and Prevention*.

[18] Hu Y., Correa A. M., Hoque A. (2011). Prognostic significance of differentially expressed miRNAs in esophageal cancer. *International Journal of Cancer*.

[19] Hiyama T., Yoshihara M., Tanaka S., Chayama K. (2007). Genetic polymorphisms and esophageal cancer risk. *International Journal of Cancer*.

[20] Liu S., An J., Lin J. (2014). Single nucleotide polymorphisms of microRNA processing machinery genes and outcome of hepatocellular carcinoma. *PLoS ONE*.

[21] Ryan B. M., Robles A. I., Harris C. C. (2010). Genetic variation in microRNA networks: the implications for cancer research. *Nature Reviews Cancer*.

[22] Bartel D. P., Chen C.-Z. (2004). Micromanagers of gene expression: the potentially widespread influence of metazoan microRNAs. *Nature Reviews Genetics*.

[23] Peng S., Kuang Z., Sheng C., Zhang Y., Xu H., Cheng Q. (2010). Association of microRNA-196a-2 gene polymorphism with gastric cancer risk in a Chinese population. *Digestive Diseases and Sciences*.

[24] Ye Y., Wang K. K., Gu J. (2008). Genetic variations in microRNA-related genes are novel susceptibility loci for esophageal cancer risk. *Cancer Prevention Research*.

[25] Shen J., Ambrosone C. B., Dicioccio R. A., Odunsi K., Lele S. B., Zhao H. (2008). A functional polymorphism in the miR-146a gene and age of familial breast/ovarian cancer diagnosis. *Carcinogenesis*.

[26] Xu T., Zhu Y., Wei Q.-K. (2008). A functional polymorphism in the miR-146a gene is associated with the risk for hepatocellular carcinoma. *Carcinogenesis*.

[27] Lin M., Gu J., Eng C. (2012). Genetic polymorphisms in microRNA-related genes as predictors of clinical outcomes in colorectal adenocarcinoma patients. *Clinical Cancer Research*.

[28] Yang H., Dinney C. P., Ye Y., Zhu Y., Grossman H. B., Wu X. (2008). Evaluation of genetic variants in microRNA-related genes and risk of bladder cancer. *Cancer Research*.

[29] Lin J., Horikawa Y., Tamboli P., Clague J., Wood C. G., Wu X. (2010). Genetic variations in microRNA-related genes are associated with survival and recurrence in patients with renal cell carcinoma. *Carcinogenesis*.

[30] Cui X.-B., Pang X.-L., Li S. (2014). Elevated expression patterns and tight correlation of the PLCE1 and NF-*κ*B signaling in Kazakh patients with esophageal carcinoma. *Medical Oncology*.

[31] Qin J.-M., Yang L., Chen B. (2008). Interaction of methylenetetrahydrofolate reductase C677T, cytochrome P4502E1 polymorphism and environment factors in esophageal cancer in Kazakh population. *World Journal of Gastroenterology*.

[32] Shang X. Q., You W. Y., Ma J. (2011). The research of correlation between single nucleotide polymorphism of hRFT1 gene and the risk of esophegeal sguamous cell carcinoma. *Journal of Nongken Medicine*.

[33] Gu L. Y., Sai L. M., Hou X. L., Chen X. C., Li F., Liu C. X. (2011). Studies on relationship between polymorphism of Esophageal cancer related gene 2 and Esophageal squamous cell cancer in Kazakh population in Xinjiang. *Journal of Nongken Medicine*.

[34] Hu J., Li L., Pang L. (2012). HLA-DRB1^∗^1501 and HLA-DQB1^∗^0301 alleles are positively associated with HPV16 infection-related Kazakh esophageal squamous cell carcinoma in Xinjiang China. *Cancer Immunology, Immunotherapy*.

[35] Christensen B. C., Avissar-Whiting M., Ouellet L. G. (2010). Mature microRNA sequence polymorphism in MIR196A2 is associated with risk and prognosis of head and neck cancer. *Clinical Cancer Research*.

[36] Cheng H.-Y. M., Papp J. W., Varlamova O. (2007). microRNA modulation of circadian-clock period and entrainment. *Neuron*.

[37] Zhao X., He X., Han X. (2010). MicroRNA-mediated control of oligodendrocyte differentiation. *Neuron*.

[38] Kocerh J., Ali Faghihi M., Lopez-Toledano M. A. (2009). MicroRNA-219 modulates NMDA receptor-mediated neurobehavioral dysfunction. *Proceedings of the National Academy of Sciences of the United States of America*.

[39] Zhang M. C., Lv Y., Qi Y. T. (2008). Knockdown and overexpression of miR-219 lead to embryonic defects in zebrafish development. *Fen Zi Xi Bao Sheng Wu Xue Bao*.

[40] Huang N., Lin J., Ruan J. (2012). MiR-219-5p inhibits hepatocellular carcinoma cell proliferation by targeting glypican-3. *FEBS Letters*.

[41] Song K.-B., Liu W.-J., Jia S.-S. (2014). MiR-219 inhibits the growth and metastasis of TSCC cells by targeting PRKCI. *International Journal of Clinical and Experimental Medicine*.

[42] Zhou Y., Du W.-D., Chen G. (2012). Association analysis of genetic variants in microRNA networks and gastric cancer risk in a Chinese Han population. *Journal of Cancer Research and Clinical Oncology*.

[43] Zeng Y., Cullen B. R. (2005). Efficient processing of primary microRNA hairpins by Drosha requires flanking nonstructured RNA sequences. *Journal of Biological Chemistry*.

[44] Sun Q., Gu H., Zeng Y. (2010). Hsa-mir-27a genetic variant contributes to gastric cancer susceptibility through affecting miR-27a and target gene expression. *Cancer Science*.

[45] Duan R., Pak C., Jin P. (2007). Single nucleotide polymorphism associated with mature miR-125a alters the processing of pri-miRNA. *Human Molecular Genetics*.

[46] Hu Z., Chen J., Tian T. (2008). Genetic variants of miRNA sequences and non-small cell lung cancer survival. *Journal of Clinical Investigation*.

